# Effect of Naphthalene Acetic Acid on Adventitious Root Development and Associated Physiological Changes in Stem Cutting of *Hemarthria compressa*

**DOI:** 10.1371/journal.pone.0090700

**Published:** 2014-03-04

**Authors:** Yan-Hong Yan, Jun-Lin Li, Xin-Quan Zhang, Wen-Yu Yang, Yan Wan, Ying-Mei Ma, Yong-Qun Zhu, Yan Peng, Lin-Kai Huang

**Affiliations:** 1 Department of Grassland Science, College of Animal Science and Technology, Sichuan Agriculture University, Ya’an, Sichuan, China; 2 College of Agronomy, Sichuan Agricultural University, Wenjiang, Sichuan, China; 3 Institute of Soil and Fertilizer, Sichuan Academy of Agricultural Sciences, Chengdu, Sichuan, China; University of Nottingham, United Kingdom

## Abstract

In order to find a way to induce rooting on cuttings of *Hemarthria compressa* cv. Ya’an under controlled conditions, a project was carried out to study the effect of naphthalene acetic acid (NAA) on rooting in stem cuttings and related physiological changes during the rooting process of *Hemarthria compressa*. The cuttings were treated with five concentrations of NAA (0, 100, 200 300, 400 mg/l) at three soaking durations (10, 20, 30 minutes), and cuttings without treatment were considered as control. Samples were planted immediately into pots after treatment. IAA-oxidase (IAAO) activity, peroxidase (POD) activity and polyphenol oxidase (PPO) activity were determined after planting. Results showed that NAA had positive effect on rooting at the concentration of 200 mg/l compared to other concentrations at 30 days after planting (DAP). Among the three soaking durations, 20 minutes (min) of 200 mg/l NAA resulted in higher percentages of rooting, larger numbers of adventitious roots and heavier root dry weight per cutting. The lowest IAAO activity was obtained when soaked at 200 mg/l NAA for 20 min soaking duration. This was consistent with the best rooting ability, indicating that the lower IAAO activity, the higher POD activity and PPO activity could be used as an indicator of better rooting ability for whip grass cuttings and might serve as a good marker for rooting ability in cuttings.

## Introduction

*Hemarthria compressa* (Poaceae), known as a warm-season perennial whip grass, mainly cultivated in southern China, south-central Florida and tropical southern Asia, is one of the most important crops in the world [1]. Recently, it is being cultivated in other parts of the world with suitable mild and humid climates. *Hemarthria compressa* (Whip grass) is used in animal feed and is effectively conserving of water logging and improving nutrient composition in soils [2]. Conventionally, whip grass is propagated by stem cuttings. However, the survival rate of mature stem cuttings was only 80% [3]. Semi-mature cuttings were used for large scale of propagation due to great demand, but the survival rate was limited by serious losses during rooting and hardening procedures [4].

The proper formation of adventitious roots at the base of stem cuttings is an important developmental phenomenon in the growth and survival of cuttings which involves the initiation of several new meristematic areas in different tissues of stem cuttings [5]. Many basic studies on adventitious root formation have been performed under *in vitro* [6]–[8] and *in vivo* [9] conditions to distinguish and delineate the successive phases of adventitious root formation and regulation, and three distinct phases of adventitious root formation in plants were found [10]–[12], i.e. induction, initiation and expression. However, different plants appeared to have different periods for the three phases in adventitious root formation. Nag *et al.* [9] reported that the period of induction, initiation and expression phase were 0–24 h, 24–72 h, after 72 h for mung bean, respectively. On the contrary, induction (0–12 days), initiation (12–14 days) and expression (14–18 days) were obtained during the adventitious rooting process of *Camellia sinensis* [13]. There is no such approach reported with grass cuttings. Based on our preliminary observation, root initiation developed at 14 days in most of the whip grass cuttings, the period of induction phase was 0–13 DAP, which was similar with *Camellia sinensis*.

In addition, adventitious root formation is generally promoted by auxin, and auxin signaling and transport has been shown to control plant root length, number of adventitious roots, root hair and root growth direction [14]. Nag *et al.* [9] reported that auxin was an essential factor for induction rather than initiation of roots in plants, which verified the hypothesis that the adventitious root formation initially occurred in two phases: an auxin-sensitive phase and an auxin-insensitive phase [15]. As a synthetic auxin, NAA is commonly used at relatively low dose to elicit auxin-type responses in cell growth, cell division, fruit setting, rooting, etc [16], [17]. The adventitious root production was increased rapidly at lower NAA concentration, while the number of roots was decreased at higher concentration [17]–[19], similar to our previous study in whip grass [20].

The activities of enzyme in the rooting zone of cuttings provided an easy, fast and reliable means of assessing cellular differentiation into roots [21]. Sato *et al.* [22] reported that a particular POD catalyzed the process of cell wall lignification during rooting in *Zinnia* cuttings. PPO catalyzes the oxidation of polyphenols and the hydroxylation of monophenols and lignification of plant cells in trees [23], [24]. Furthermore, an auxin-induced change in POD and IAAO occurred during the rooting processes [9]. But, the responses of enzyme activities to auxin in forage grass were unclear. Therefore, we examined the effects of NAA concentrations and soaking durations on enzyme activities in the rooting zone during the induction phase of adventitious root formation and rooting response in whip grass stem cuttings. These research results may be used as an indicator of rooting ability and lay a theoretical foundation for the improvement of whip grass rooting.

## Materials and Methods

### Plant materials and treatments

The cuttings of *H. compressa* (“Ya’an” cultivar) were selected from the Farm of Sichuan Agricultural University, which have been released by the China Forage Registration Committee, and have been widely used in the Yangtze River and played an important role in animal husbandry. Experimental stem cuttings of 15–16 cm length, 3.0 mm diameter and with 3 nodes were obtained from vigorously growing whip grass at the elongation stage on 9 May of 2011 and 2012. Leaves were removed from the cuttings, and the cuttings were immersed in a solution of fungicide 0.5% Bavistin [Methyl N- (1Hbenzoimidazol-2-yl) carbamate] for 15 min and thoroughly rinsed with distilled water. The basal ends (5 cm) of each cutting were then soaked in different concentrations (0, 100, 200, 300, and 400 mg/l) of NAA for different durations (10, 20, 30 min) and cuttings without treatment were considered as control. The treatments were based on the results from preliminary trials in the laboratory. There were three replicates of 20 cuttings in each treatment, which were planted immediately in pots (30 cm diameter, 45 cm deep) filled with a sterilized mixture of sand and loamy soil (1:2, v:v). The pots were then incubated in a controlled growth chamber for 30 days (25±2°C, 16 h photoperiod with cool, white fluorescent lamps and 65% relative humidity). Watering was applied every two days.

### Scoring of data in rooting experiment

Ten cuttings were taken randomly from each treatment at 30 DAP for morphological analysis in three replicates. Rooting percentage, numbers of adventitious roots (longer than 2 cm) per cutting and root dry weight per cutting were recorded.

### Physiological investigation

There were three replicates of ten cuttings in each treatment for physiological analysis. The physiological investigations on the basal part (about 0.5∼1.0 cm of the rooting zone) were carried out, cut up and mixed at 10 DAP (induction phase).

### Extraction for enzyme assay

Fresh tissue (0.5 g) was weighed from the above mixed samples in each treatment with three replicates, frozen in liquid nitrogen and stored at –80°C. The frozen sample was ground in a mortar on ice with 10 ml of 100 mM pre-cold phosphate buffer (pH 6.0) containing 1 % (w/v) dithiothreitol for enzyme extract. The homogenate was centrifuged at 10,000 rpm for 20 min at 4°C and then the pellet was discarded and the supernatant was used for enzyme assay.

### IAAO assay

The reaction mixture was made of the 0.5 ml enzyme extract, 1.5 ml of 100 mM potassium-phosphate buffer (pH 6.0), 1.0 ml of 1 mM MnCl_2_, 1.0 ml of 1 mM 2, 4-dichlorophenol and 1.0 ml of 1 mM IAA. Assay was conducted at 30±0.5°C for 30 min. Then, 0.08 ml of 0.5 M FeCl_3_ and 3.92 ml of 35% perchloric acid were added to the above enzyme mixture (2 ml). IAA degradation was determined by measuring the absorbance at 530 nm at 35±0.5°C for 30 min in the dark [25]. IAAO activity is represented by the amount of IAA degraded (µg) starting from 1 mg initial protein in 1 h. Measurements were performed in three replicates.

### POD assay

The activity of POD was determined according to the method of Li *et al.* [26] based on the oxidation of guaiacol using H_2_O_2_. The enzyme extract (0.05 ml) was added to 3 ml reaction solution to start reaction and the absorbance was monitored at 470 nm for 2 min. POD activity was quantified by the amount of tetraguaiacol formed. The reaction solution was made by mixing 50 ml 100 mM cold phosphate buffer (pH 6.0), 28 µl guaiacol, then, heated and stirred until guaiacol was completely dissolved, finally, 19 µl 30% H_2_O_2_ was added after it was cooled and mixed evenly.

### PPO assay

The activity of PPO was determined according to the method of Li *et al.* [26] with modification. The 1 ml reaction mixture contained 250 µl of the enzyme extract and 0.1 M phosphate buffer (pH 6.0). Each sample was aerated for 2 min in a small test tube followed by the addition of 0.2 M catechol as the substrate. PPO activity was expressed as changes in absorbance at 420 nm min^−1^ g^−1^ FW.

### Statistical Analyses

Means of three replications were calculated. All data were analyzed using the two-way analysis of variance and the least significance difference (LSD) test at p  =  0.05 [27] for comparisons between NAA dosages and soaking durations using the SAS program version 9.1 (SAS Institute, Cary, NC).

## Results

### Effect of NAA-treatment on rooting

**Figure 1:**
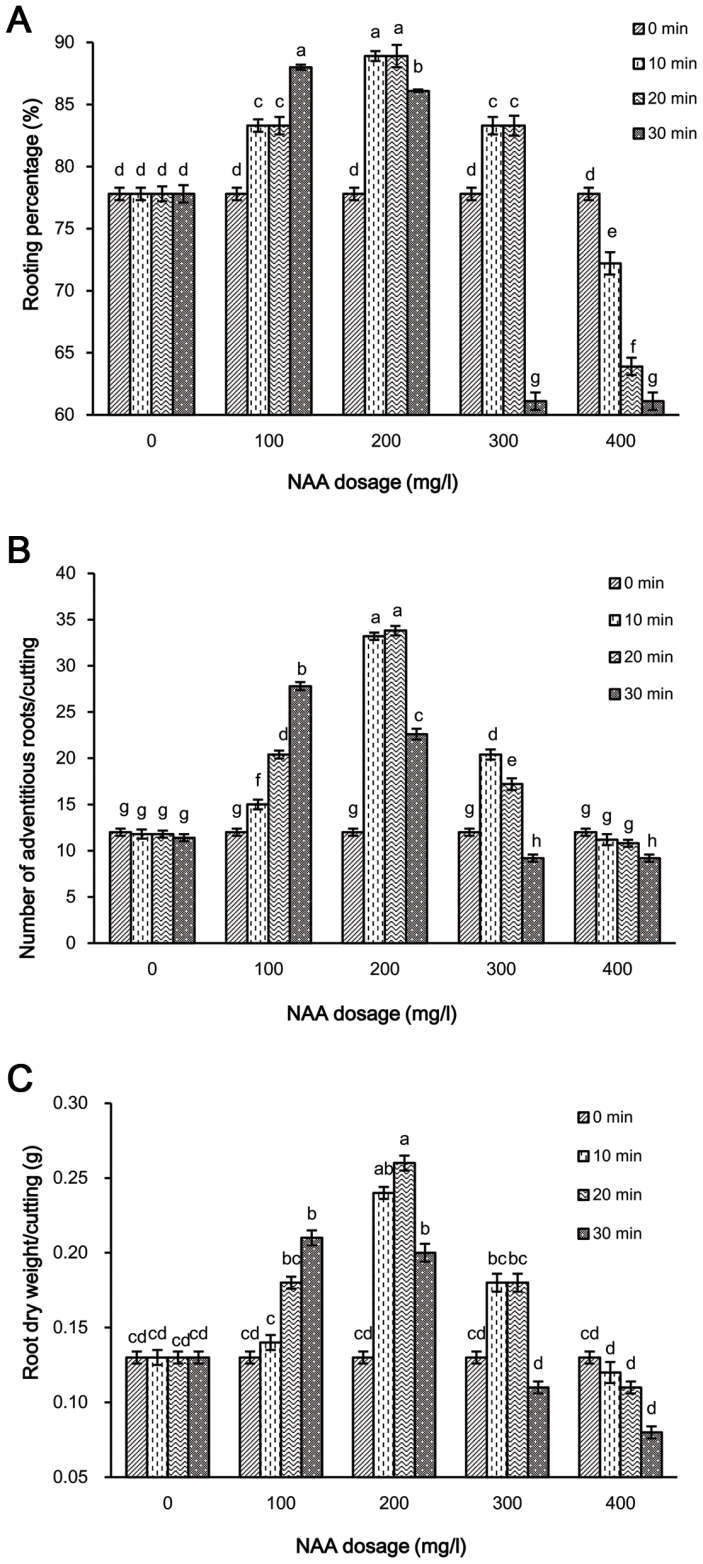
Rooting response in whip grass cuttings under treatment of NAA dosage and soaking duration in 2011. Comparative graph of rooting percentage (A), number of adventitious roots (longer than 2 cm) per cutting (B) and root dry weight per cutting (C). 0 min soaking duration of different NAA dosages (0 mg/l, 100 mg/l, 200 mg/l, 300 mg/l and 400 mg/l) was without treatment and considered as control. Different small letters mean significant differences among treatments of NAA dosage and soaking duration at 30 DAP at α = 0.05. Values represent a mean of 3 replicates with SD. LSD Test.

Rooting characteristics were influenced by different NAA dosages and soaking durations for explants excised in the year 2011 and 2012 (Figure 1, Table 1). Interestingly, the treatments with lower dosages NAA (100 mg/l and 200 mg/l) increased rooting percentage, number of adventitious roots per cutting and root dry weight per cutting. Maximum rooting ability was observed in the 200 mg/l NAA treatment, whereas higher dosage NAA (400 mg/l) suppressed these parameters. However, rooting ability was not affected by soaking duration in the non-NAA condition. The rooting percentage, number of adventitious roots per cutting and root dry weight per cutting were significantly increased with the increase in soaking duration of 100 mg/l NAA at 30 DAP. When treated with 300 mg/l NAA, the maximum of the root parameters studied at 30 DAP, was observed in cuttings treated during 10 min soaking. However, the cuttings treated with 200 mg/l NAA ×20 min soaking duration showed higher percentages of rooting as well as larger numbers of adventitious roots and heavier root dry weight per cutting.

**Table 1:** Rooting response in cuttings of whip grass under treatment of NAA dosage and soaking duration in 2012.

		NAA dosage (mg/l)				
Characteristics	Soaking duration (min)	0	100	200	300	400
Rooting percentage (%)	0	80.9±0.50e	80.9±0.50e	80.9±0.50e	80.9±0.50e	80.9±0.50e
	10	80.9±0.51e	88.9±0.53d	97.2±0.42a	86.7±0.76d	78.1±0.94fg
	20	80.8±0.63ef	91.7±0.74c	97.2±0.92a	86.7±0.80d	78.1±0.71fgh
	30	80.7±0.72ef	94.4±0.25b	94.4±0.12b	75.3±0.71gh	72.6±0.72h
Number of adventitious roots/cutting	0	12.2±0.39g	12.2±0.39g	12.2±0.39g	12.2±0.39g	12.2±0.39g
	10	12.2±0.50g	15.2±0.52f	34.8±0.40a	21.2±0.55d	10.2±0.60hi
	20	11.6±0.38gh	20.8±0.43d	35.4±0.51a	17.6±0.63e	9.0±0.38ij
	30	11.2±0.39gh	28.6±0.44b	23.4±0.59c	8.2±0.38jk	7.0±0.39k
Root dry weight/cutting (g)	0	0.24±0.01cde	0.24±0.01cde	0.24±0.01cde	0.24±0.01cde	0.24±0.01cde
	10	0.21±0.02cde	0.26±0.02cd	0.34±0.01ab	0.3±0.01b	0.16±0.01ef
	20	0.19±0.02de	0.29±0.02bc	0.4±0.02a	0.28±0.01bc	0.15±0.01ef
	30	0.17±0.01e	0.32±0.01b	0.3±0.01b	0.14±0.01ef	0.07±0.01g

Data were recorded at 30 DAP. Value represented a mean of three replicates with SD of each treatment, which consisted of 10 cuttings. 0 min soaking duration of different NAA dosages (0 mg/l, 100 mg/l, 200 mg/l, 300 mg/l and 400 mg/l) was without treatment and considered as control. Within the same characteristics, means followed by the same letters are not significantly different at P<0.05 according to LSD tests.

### Effect of NAA-treatment on IAAO activity

**Figure 2:**
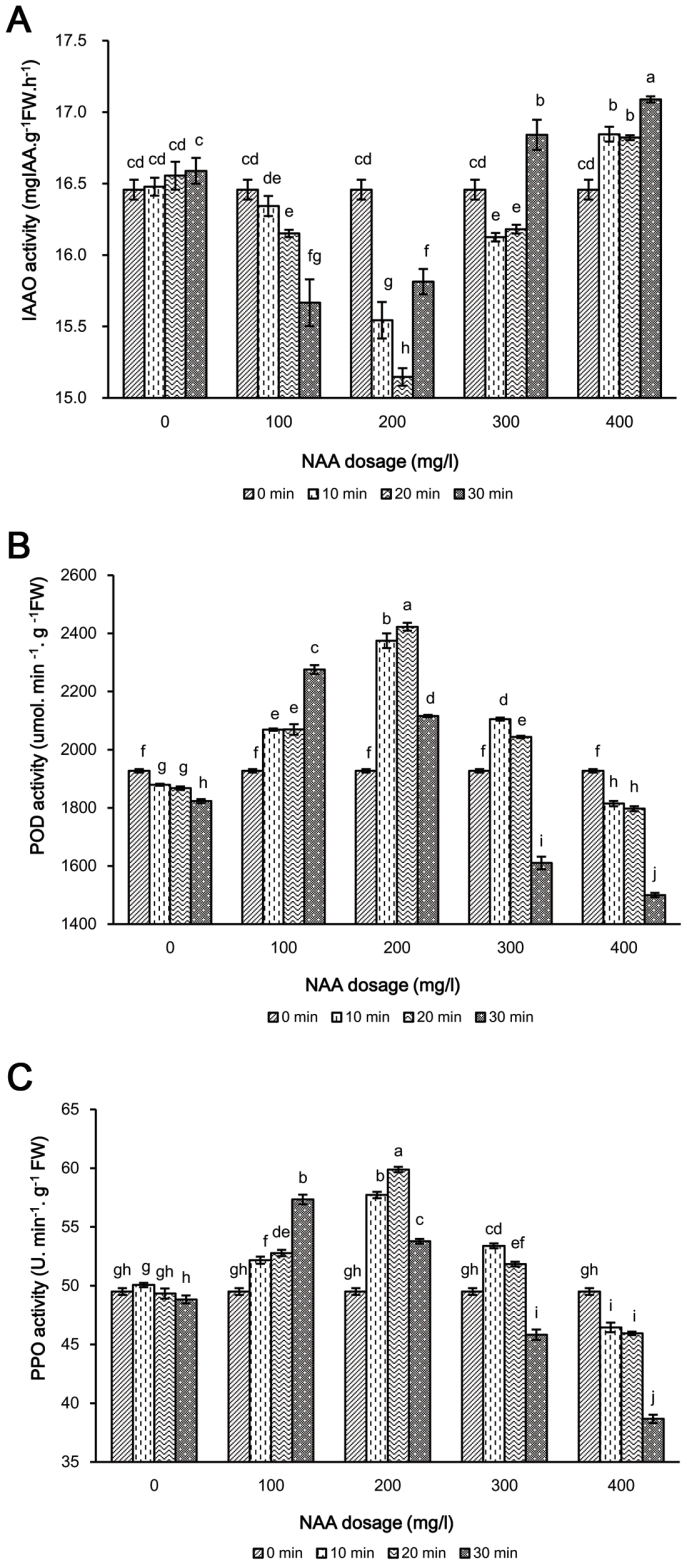
Effect of NAA dosage and soaking duration on some enzyme activity in whip grass cuttings in 2011. Comparative graph of IAAO activity (A), POD activity (B) and PPO activity (C). 0 min soaking duration of different NAA dosages (0 mg/l, 100 mg/l, 200 mg/l, 300 mg/l and 400 mg/l) was without treatment and considered as control. Different small letters mean significant differences among treatments of NAA dosage and soaking duration at 10 DAP (induction phase) at α = 0.05. Values represented a mean of 3 replicates with SD. LSD Test.

The IAAO activity was affected by NAA dosages for different soaking durations of cuttings excised from plants in 2011 and 2012 (Figure 2A, Table 2). The IAAO activity was significantly decreased in the cuttings treated with 100 mg/l and 200 mg/l NAA compared to the control, and the maximum reduction in IAAO activity was observed in 200 mg/l NAA treatment. However, IAAO activity was significantly increased in cuttings treated with 400 mg/l NAA. IAAO activity at 10 DAP was significantly decreased with the increase in soaking duration of 100 mg/l NAA. Among the different soaking durations of 300 mg/l NAA, the minimum value of IAAO activity at 10 DAP was obtained in cuttings treated during 10 min soaking. However, the most remarkable inhibition occurred at 200 mg/l NAA ×20 min soaking duration, and the IAAO activity values were 8.51% and 4.03% lower compared to the control in 2011 and 2012, respectively.

**Table 2:** Effect of NAA dosage and soaking duration on some enzyme activity in whip grass cuttings in 2012.

		NAA dosage (mg/l)				
Characteristics	Soaking duration (min)	0	100	200	300	400
IAAO (mg IAAg^−1^FW.h^−1^)	0	16.02±0.01f	16.02±0.01f	16.02±0.01f	16.02±0.01f	16.02±0.01f
	10	16.07±0.01f	15.87±0.07g	15.50±0.04j	15.75±0.07gh	16.26±0.03d
	20	16.12±0.01ef	15.78±0.06g	15.40±0.02k	15.85±0.09g	16.38±0.08c
	30	16.23±0.03de	15.58±0.06ij	15.64±0.08hi	16.52±0.07b	16.74±0.04a
POD (μmol.min-1.g-1FW)	0	2410.69±1.63h	2410.69±1.63h	2410.69±1.63h	2410.69±1.63h	2410.69±1.63h
	10	2395.83±5.52h	2443.75±21.94g	3237.50±6.88b	2710.42±7.29e	2141.67±6.14j
	20	2233.33±6.04i	2627.08±9.10f	3668.75±14.79a	2636.81±3.02f	2134.03±14.08j
	30	2152.08±11.58j	3172.92±9.07c	2745.83±12.33d	2010.42±14.08k	1960.42±17.07l
PPO (U.min-1.g-1FW)	0	59.17±0.27g	59.17±0.27g	59.17±0.27g	59.17±0.27g	59.17±0.27g
	10	57.94±0.09h	60.44±0.38ef	68.0±0.24b	61.39±0.89de	55.06±0.27i
	20	56.89±0.21h	60.89±0.12ef	69.61±0.17a	60.28±0.32fg	54.56±0.37i
	30	55.44±0.39i	62.83±0.42c	62.0±0.65cd	51.33±0.09j	51.33±0.49j

Data were recorded at 10 DAP. Value represented a mean of three replicates with SD of each treatment, which consisted of 10 cuttings. 0 min soaking duration of different NAA dosages (0 mg/l, 100 mg/l, 200 mg/l, 300 mg/l and 400 mg/l) was without treatment and considered as control. Within the same characteristics, means followed by the same letters are not significantly different at P<0.05 according to LSD tests.

### Effect of NAA-treatment on POD activity

POD activity was influenced by NAA dosages for different soaking durations of cuttings excised from plants in 2011 and 2012 (Figure 2B, Table 2). 100 mg/l and 200 mg/l NAA treatments caused a significant increase in POD activity, and the most remarkable promotion of POD activity occurred at 200 mg/l NAA treatment, but 400 mg/l NAA treatment caused a significant reduction compared to the control. POD activity was promoted significantly with increasing soaking duration for 100 mg/l NAA. Additionally, the highest POD activity was for the 20 min soaking duration, followed by 10 min, and then 30 min under 200 mg/l NAA, which were significantly higher than the control by 25.73%, 23.24% and 9.80% in 2011, and 52.19%, 34.30% and 13.90% in 2012, respectively. On the contrary, POD activity was reduced with increasing soaking durations under 300 mg/l and 400 mg/l NAA treatments.

### Effect of NAA-treatment on PPO activity

PPO activity was significantly affected by the NAA dosages for different soaking durations of cuttings excised from plants in 2011 and 2012 (Figure 2C, Table 2). PPO activity was significantly increased in the cuttings treated with 100 mg/l and 200 mg/l NAA, and the highest PPO activity was observed in 200 mg/l NAA treatment. However, PPO activity was significantly decreased in cuttings treated with 400 mg/l NAA. Additionally, the PPO activity values were maximized when soaked at 100 mg/l NAA for 30 min soaking duration and soaked at 200 mg/l NAA for 20 min soaking duration in both years. The most remarkable promotion occurred at 200 mg/l NAA×20 min soaking duration, respectively 20.99% and 17.64% higher compared to the control in 2011 and 2012.

## Discussion

This study showed that adventitious root formation was influenced by NAA dosage and soaking duration for cuttings obtained in 2011 and 2012, but the value of rooting characteristics was higher for the 2012 cuttings than for the 2011 cuttings. This may be because the stem cuttings in 2012 were more mature. Similarly, Dick and Magingo [28] reported that rooting ability of cuttings varied among clones and node positions. Of the NAA dosages tested, lower dosage of NAA caused a significant increase in rooting ability as compared with the control, and 200 mg/l was found to be the best at enhancing rooting ability, while higher doses of NAA caused a significant decrease in root formation. This verified Hentig and Gruber’s [29] report that hormonal doses could induce the best rooting when being just below the toxic level. Similar results were obtained in Sun and Hong’s [17] research, which found that lower concentrations of NAA (1.0 mg/l and 2.0 mg/l) in the callus induction medium had a stronger effect on successive plant regeneration of the halophyte *Leymus chinensis* than higher concentrations. In contrast, the range of effective NAA concentration was much lower compared with our experiment, this may be due to the difference between cuttings cultured both *in vitro* and *ex vitro* [30].

Furthermore, auxin is widely applied in the vegetative propagation of various plants [31], [32], and there are many deviations in the range of effective NAA dosage on cuttings of different plant species. Cui and Mao [33] reported that the best way to apply auxin was to dip quickly with 3,000 mg/l NAA for root growth and cutting propagation of *Zizphus spinosus*. In the case of teak stem cuttings, the maximum number of roots per cutting was produced by quick dipping in 4,000 mg/l NAA [22]. In the present study, treatment with 200 mg/l NAA for 20 min soaking duration produced the highest rooting percentage and the maximum number of adventitious roots per cutting in whip grass, followed by treatment with 200 mg/l NAA for 10 min soaking duration (Figure 1, Table 1). This showed that the effective NAA dosage in cuttings of trees was higher than that of grass cuttings.

The stimulatory effects of NAA on rooting of plant stem cuttings have been reported by many researchers, and promotion of adventitious root formation has been considered as one of the characteristics of auxin [13]. There is considerable evidence that indole-3-acetic acid (IAA) is involved in adventitious root formation. Liu and Reid [34] reported that high levels of IAA were associated with the promotion of adventitious root. In *Arabidopsis*, high concentrations of exogenous IAA triggered pericycle cells adjacent to protoxylem poles to become part of a lateral root primordium [35]. Kowalczyk *et al.* [36] reported that the IAA levels increased after treatment in NAA solution of mesocotyl and coleoptile segments of maize. In addition, IAAO activity might play a crucial role in regulating endogenous IAA levels [9], and the relationship between the levels of IAA and IAAO activity is negatively correlated [37]. In the present study, the lowest IAAO activity during the induction period was obtained in the cuttings treated with 200 mg/l NAA for 20 min soaking duration (Figure 2A, Table 2), resulting in the highest rooting ability. The results indicated that the lower IAAO activity during the induction period in lower NAA dosage treated cuttings appeared to be responsible for better development of adventitious roots, possibly being related to higher endogenous IAA content [9]. Similarly, the soybean hypocotyls of NAA-treated cuttings grew significantly higher numbers of adventitious roots with an increase in endogenous IAA levels that corresponded with a decrease in IAAO activity examined [38].

The formation of adventitious root involves the process of redifferentiation during which predetermined cells switch from their morphogenetic path to act as mother cells for the root primordia [39]. Among these changes, POD activity is known to be involved in auxin metabolism as well as in lignification process in the cell wall synthesis an obligatory step in root formation [13], [23]. The present results showed that an increase in POD activity was observed at the induction phase in 100 mg/l and 200 mg/l NAA-treated cuttings, and the highest POD activity in the cuttings treated with 200 mg/l NAA for 20 min soaking duration was consistent with the rooting ability (Figure 2B, Table 2), indicating that POD activity was an indicator of better rooting ability and might serve as a good marker for rooting ability in cuttings [40]. Likewise, auxin-induced changes in POD during the rooting process have also been reported [38], [41]. These results postulated that the POD acts as antioxidants, thereby protecting IAA from oxidation and plant tissue from oxidative stress due to wounding.

In addition, PPO catalyzes the oxidation of polyphenols and the hydroxylation of monophenols and lignification of plant cells during the rooting process [24]. It is positively correlated with lignin content in both shoot and fruit of eggplant, and the higher amount of PPO and lignin contents improved rooting and resistance to biotic stress [25]. Our results showed that the PPO activity was increased with lower NAA dosage, which was consistent with Rout’s data [16], indicating that the increase in the activity of PPO during the induction phases might be associated with better rooting ability in treated cuttings. The present results also showed that PPO activity was highest in 200 mg/l NAA for 20 min soaking duration treated cuttings, which was significantly higher than that of 200 mg/l NAA for 10 min soaking duration and 100 mg/l NAA for 30 min soaking duration (Figure 2C, Table 2). These results suggested that PPO activity metabolism of whip grass cuttings was sensitive to NAA dosage and soaking duration, similar to the results of Hunt *et al.* [42] and James [43], which found that the concentration of auxin and the timing of its application were key factors in successful rooting and associated physiological changes.

## Conclusion

In our study, 100 mg/l and 200 mg/l NAA increased rooting ability, POD and PPO activity, but decreased IAAO activity during the rooting induction phase in whip grass cuttings. The changes in physiological parameters by the use of NAA seemed related to the changes of morphological traits, as NAA induced adventitious roots, with a concomitant increase in dry weight of the roots per cutting. Higher activities of POD and PPO and lower IAAO activity in the cuttings might be among the critical factors to improve rooting, and to prevent membrane damage. The present studies indicated that 200 mg/l NAA for 20 min soaking duration had a significant effect on root regeneration in whip grass cuttings. Thus, these results would be useful for mass-scale propagation and conservation of whip grass, future experiments should be conducted to examine the validity of these results with other forage grass.
